# Putting things in and taking them out of containers: a young child's interaction with objects

**DOI:** 10.3389/fpsyg.2023.1120605

**Published:** 2023-05-23

**Authors:** Chihiro Nishio, Hikaru Nozawa, Hiroe Yamazaki, Kazutoshi Kudo

**Affiliations:** ^1^Department of Psychology, Chukyo University, Nagoya, Japan; ^2^Graduate School of Arts and Sciences, The University of Tokyo, Tokyo, Japan; ^3^Faculty of Education, Tokyo Gakugei University, Tokyo, Japan; ^4^Interdisciplinary Information Studies, The University of Tokyo, Tokyo, Japan

**Keywords:** child-object interaction, natural observation, development of organized behavior, carrying objects, social interaction

## Abstract

**Introduction:**

How does the behavior of putting things away (*putting them in*) in a container and using them again (*taking them out*) develop in young children? Though object interaction is one of the most examined topics in child development, research on organized behavior with various objects and containers at home is lacking. Rather than conducting experiments on young children's interactions with objects, this study focused on natural child–object interaction in the home.

**Methods:**

We conducted a case study on a young child's natural interaction with objects at home, focusing on when the child puts them in or takes them out of a container (the shelf, the cabinet, or the box). The study took place over 2½ years.

**Results:**

The behaviors of putting many objects in a container and taking them out appeared at 9 months old. After acquiring the skill of walking, the child carried the objects using bags. Putting objects in and taking them out was embedded in the locomotion, and the child prepared the containers of toys before play. Pulling as many objects out as possible became rare after 19 months of age. Taking objects out became more appropriate in that context. The child brought out the container before the activity and put things away afterward.

**Discussion:**

Based on these findings, the development of organized object interaction as well as the anticipation and significance of the naturalistic longitudinal observations are discussed.

## 1. Introduction

Examining infant interactions with an object is one way to learn about how they develop. In particular, there is abundant literature on infant development focusing on object interactions. Previous studies relating to infant–object interaction span several topics such as the concept of object and object permanence, which develops gradually during the sensorimotor stage of cognitive development (Piaget, 1954), the development of anticipation and motor control when grasping objects (von Hofsten and Rönnqvist, 1988; Claxton et al., 2003), and the development of triadic interaction (between an infant, an adult, and an object) and joint attention (Bakeman and Adamson, 1984; Little et al., 2016). These studies revealed cognitive and social development through infants' interactions with objects. Recent studies have focused more on the variety of objects in the natural environment. Clerkin et al. (2017) recorded the infant-perspective view using a head-mounted camera to examine the everyday experiences of daily-life objects and related word learning. Herzberg et al. (2021) examined infant interactions with objects at home and demonstrated the variety of objects that infants engaged with. These studies focus on interaction's effect on development with various objects in everyday environments.

Recent studies also focus on walking and infant–object engagements and suggest that carrying objects can lead to behavioral changes (Karasik et al., 2012, 2013; Cole et al., 2015; Toyama, 2018). When an infant starts walking, carrying objects becomes much more frequent (Karasik et al., 2012, 2013). When they bring the object to their caregivers, walking infants receive different verbal responses than crawling infants. The caregivers recognize them as competent in carrying objects around. They elicit new actions from the walking infants by asking them to take the object to another place (Karasik et al., 2013). Infants then become more demanding of caregivers; they make the caregivers watch them using objects (Toyama, 2018). These studies demonstrate that carrying objects instigates, starts, or ends a social event.

Though carrying objects is common in infant behavior and leads to an infant starting a new social event, Cole et al. (2015) indicate that most infant object carrying does not have any specific goals. As Orrmalm (2020) pointed out, infants frequently spread out objects and scatter them around. Infant walking and object carrying are exploratory. Then, when and how does the exploratory behavior develop into organized behavior, such as bringing the toys to an open space and putting them away after the play session? Nonaka and Sasaki (2009) longitudinally observed that a toddler repeatedly gathered toy blocks and put them into the toy box at home. The child then adjusted their position near the box and gathered the blocks while using its lid as a tray to facilitate putting the blocks into the box. The toddler's behavior became flexible, and the object interaction became organized. In the long term, carrying objects and taking them out of and putting them into the container might be adjusted into a social event in the daily environment. Thus, for behavior such as preparing or cleaning up to emerge, infants must be able to anticipate the structure of the upcoming event and adapt their behavior to it.

Human environments are structured and aligned with daily activities; accordingly, there are functionally different places for cooking, eating, relaxing, or sleeping. A certain behavior such as eating is typically patterned in characteristic ways, and children are expected to be socialized into the routine (Baggs, 2021). Though playing is less structured behavior compared to such routines, there are typically places or containers for toys to be set as open spaces in the home, yet these areas have multiple functions, and scattered toys hinder other activities. Because of the nature of the daily environment, toys or objects that attract infants may be set on a cabinet in the corner of the living room or put into boxes on a shelf. Playing with these objects is necessarily accompanied by carrying objects and interacting with objects and containers.

In previous developmental studies, activities such as cleaning up toys have been considered from the perspective of social rules or compliance with the parents (Power and Parke, 1986; Gralinski and Kopp, 1993; Kochanska et al., 2001). However, these studies did not examine infant interactions with objects in detail, and the developmental process that object interaction and carrying objects would embed into a social context thus remains unknown. Learning about objects and containers and what they do should be accompanied by the development of this behavior because toys and daily goods vary significantly in size and shape, and the range and combination of these objects and containers are enormous. What these environments—including objects or others—offer us are called affordances (Gibson, 1986). Affordance refers to the meaning and possibility of action that animals or humans perceive in a certain environment. Specifically, infants learn what they can do with a certain object and container. Moreover, it can be assumed that infants learn by observing what others do in the home. To examine the development of organized behavior, attention should be paid to affordances and the influence of others.

Some studies on tool use development have revealed the process by which infants learned the affordances of specific objects and how exploratory actions converge into a certain pattern of tool use. Examples include a spoon or fork (Nonaka and Goldfield, 2018) as well as zippers and lids (Rachwani et al., 2021). These studies focus on the process of development of functional behavior in a specific context such as eating or opening a container. Behavior such as bringing toys before playtime and putting them away is less goal-oriented than tool use in a specific daily scene and should happen in a much more varied context in everyday life.

This study aimed to examine the development of organized object interactions in the context of everyday activities; in particular, the process of the development of carrying objects and putting them into and taking them out of containers becomes embedded in the structure of social events. When infants scatter their toys, caregivers will clean them up. However, Nonaka and Sasaki (2009) indicated that organized behavior such as cleaning up, gathering blocks together, and putting them in the box emerged around the ages of 18–24 months. To achieve our goal, we focused on the daily activity of an infant/toddler taking objects out of them and putting them into containers, shelves, or cabinets. We conducted a longitudinal observation of one child to reveal how object interaction with such containers changed as well as how and when the behavior of putting objects away emerged.

This is a single-case study based on a longitudinal observation at a home. Experiments in the laboratory cannot examine the complex process of development of infant–object interactions in daily life because it is difficult to reproduce daily environments. These particular environments include familiar objects (not novel toys) surrounded by consistent layouts of the furniture and rooms and where daily events such as eating meals or playing begin and end. Actions such as picking up an object, carrying it, and then handing it to others are embedded in natural environments that include others. Such nested actions emerge in the interactions between infants and people in the same environment. For these reasons, we conducted a longitudinal study that continuously observes natural behaviors embedded in daily life as an exploratory and emergent study. Although this is a case study, we believe that the generality and reproducibility of the findings obtained may be expanded on through additional case studies in future.

## 2. Materials and methods

### 2.1. Participants and procedures

**Figure 1:**
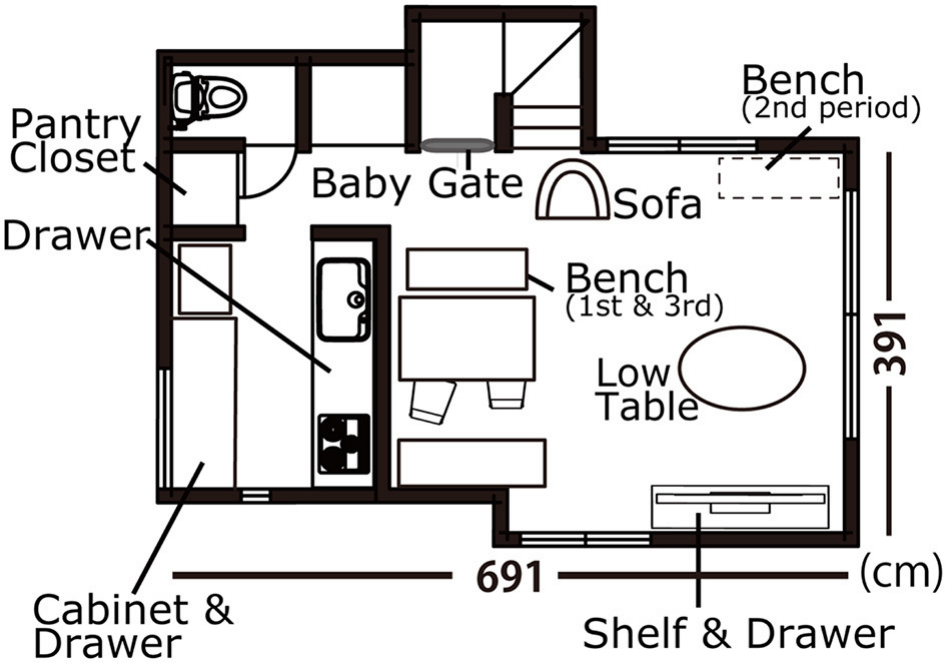
Layout of the living room and kitchen. The open cloth box (toy box), the puzzle box, the block tray, and the green block box with which R frequently engaged were put under the storage bench. The storage bench was moved to the corner of the room during the second period. The storage bench contained picture books as well. Small-size picture books were also on the shelf of the TV units. The drawer of the TV units contained toys, crayons, and paper.

A family acquainted with the researcher living in Tokyo participated in this study. “R” was the second-born daughter of the family, and she had a sister who was 6 years older than her. Figure 1 shows the map of R's house. Her parents were instructed to video-record their living room every week for 1 to 2 h each time. A small video camera with a wide-angle lens was set up to cover the entire living room. Recordings were often made in the afternoon; however, we did not ask the parents to record videos specifically on the same day and at the same time, considering convenience and feasibility for long-term participation. Videos were collected from the time when R was 6 months old until she was 32 months old. After R started crawling at 7 months, another video camera was set in the kitchen. The researcher visited the home every 2 weeks to observe R's behavior directly and note the environmental changes, including the object type. R's family members engaged in daily activities such as doing chores, relaxing, playing, reading books, or watching TV. This study was approved by the University of Tokyo Ethics Committee.

### 2.2. Data analysis

We analyzed the data from the 7-month-old point—when R reached the storage bench by belly-crawling—to the 32-month-old point. The total length of the recording time was 242 h. The scenes that included R's interactions with objects being put in or taken out of concavities (hollowed out/curved spaces) or enclosures such as cabinets, shelves, boxes, and containers were extracted. Cups or bowls used during mealtime were excluded from analysis because the focus of this study was to examine the effect on infant interactions with objects concerning functionally different places. When R was fed, she sat on a chair, and the activity did not include locomotion between functionally different places. The total number of cases was 428. Moving forward, we will use the word “container” to refer to the concavities or enclosures, such as the cabinets, shelves, stockers, or boxes, in this research.

The observed period was broken down into three parts. The previous studies on behavior around the time of walking onset focused on the period of around 13–19 months (Adolph et al., 2012; Cole et al., 2015; Hoch et al., 2021). First, we split the period into three at the walking onset. Specifically, we had the first period, which was 7 months from the 1st day R crawled to reach the bench stocker in which picture books and an open container with toys were located (7m23d−12m26d). Next was the second period, which was 6 months from the first day R walked five consecutive steps (12m29d−18m24d). Finally, the rest of the period for about 1 year after the second period (19m1d−32m21d) was set as the third period.

Using the Datavyu coding software, version 1.3.7 (Datavyu Team, 2014), the scenes of putting things into and/or taking them out of the container were separated as one object interaction episode based on the following criteria. The object interaction episode was defined as when R put multiple or single objects into the container and/or took the objects out of it. The beginning of the episode was set at her touch of the detached object or container. When she picked an object up and put it into something, the beginning of the episode was set at the first touch of the object. If she opened the cabinet or drawer before she touched an object inside, the beginning was set at the first touch of the cabinet or drawer. The end of the event was set based on the following criteria. When she moved more than three steps without carrying anything, the end was set at the time she departed. When she took her hands away from the object or container and did not touch them for more than 20 s, the end of the event was set just after the last touch of the object or container. If she took something out of the container and kept holding it or carrying it, it was regarded as one event. The number of total events in the first period was 144; the second period had 174, and the third period had 110.

All object and container types that R engaged with were noted. We identified unique objects that she frequently handled, though we did not identify objects with multiple pieces such as books on the shelf, blocks in the toy box, tissue paper in the box, or kitchen bowls in the cabinet as Herzberg et al. (2021) did. When she successively engaged with multiple objects or containers, all of them were noted. Whether an event included carrying or no carrying was subsequently coded. Whether an event included social interactions were also coded based on the following criteria: when she passed or showed objects to others, when others passed or showed objects to her, when she and others played with objects, or when they arranged objects together. Finally, the duration of each event was calculated.

We coded the duration of the object interaction episodes, the types of objects R engaged with, carrying objects, and social interactions. Detailed case studies were conducted using illustrations for selected episodes that could show typical changes in container types over the three time periods.

## 3. Results

### 3.1. Overview

**Figure 2:**
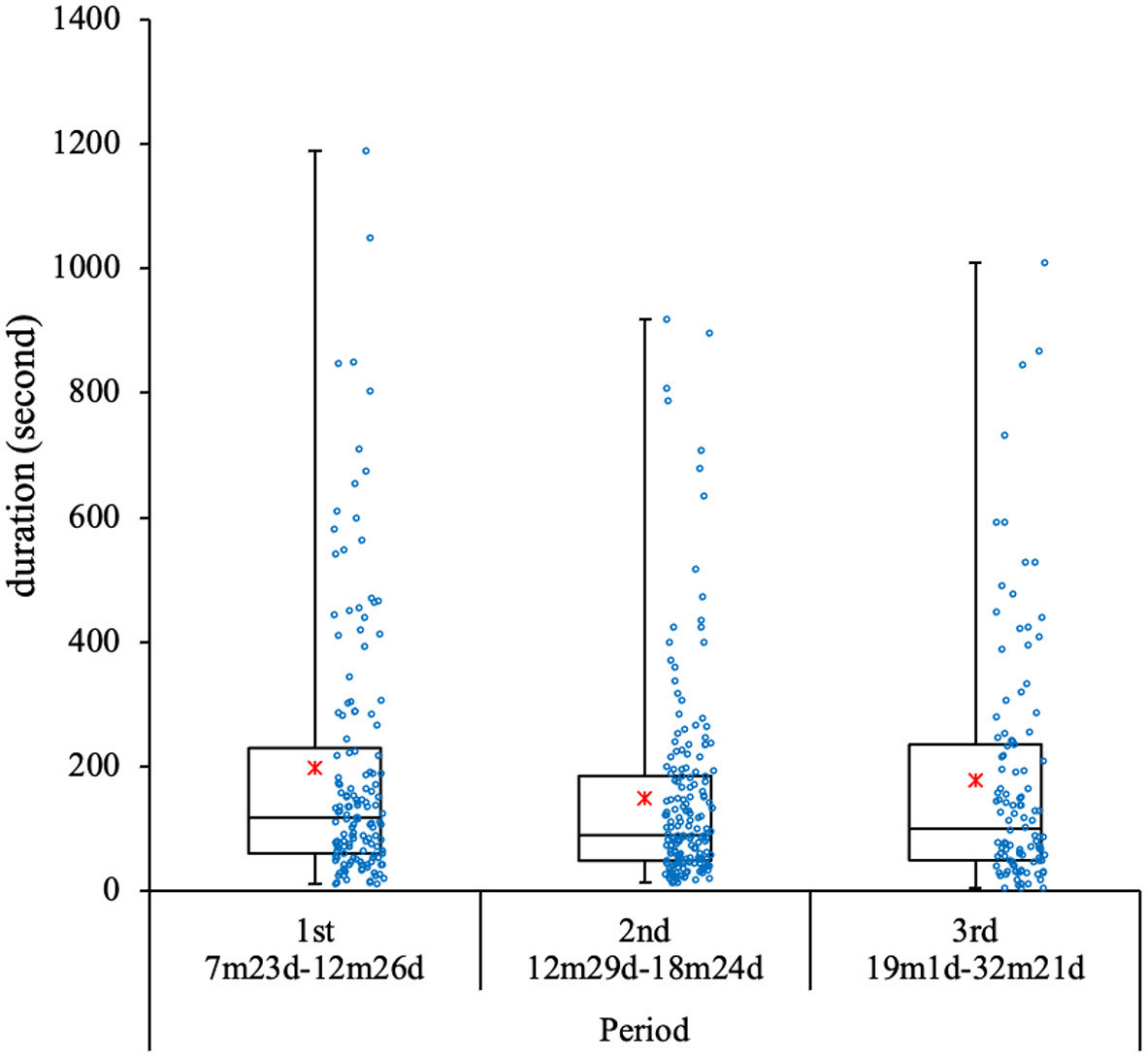
Duration of each event for three periods. Each dot represents one event.

Figure 2 shows the duration of each event for the three periods. The average duration of each event was 172.1 s (*SD* = 192.6). The average duration of events was 196.9 s (*SD* = 159.9) for the first period, 149.1 s (*SD* = 145.5) for the second period, and 176.1 s (*SD* = 183.0) for the third period. As the distribution was not normal, we conducted a Kruskal–Wallis test. Subsequently, the results showed no significant differences among the three periods.

Table 1 shows the frequency of carrying for the three periods. The difference between the periods was significant with the following: χ^2^(2, N = 428) = 155.93, *p* < 0.001. Except for one instance, carrying did not occur in the first period. In the second period, 58.6% of the total events included carrying, while in the third period, 70% of the events included carrying.

**Table 1:** Frequency of carrying objects in each period.

**Period**	**1st (7m23d−12m26d)**	**2nd (12m29d−18m24d)**	**3rd (19m1d−32m21d)**	**Total**
Without carrying	99.3% (143)	41.4% (72)	30% (33)	57.9% (248)
Carrying	0.7% (1)	58.6% (102)	70% (77)	42.1% (180)

Table 2 shows the frequency of social interactions. The difference between the periods was significant with the following: χ^2^(2, N = 428) = 31.422, *p* < 0.01. In the first period, 5.6% of the total events included social interactions, while in the second and third, 30% of the total events included social interactions.

**Table 2:** Frequency of social interactions in each period.

**Period**	**1st (7m23d−12m26d)**	**2nd (12m29d−18m24d)**	**3rd (19m1d−32m21d)**	**Total**
No interaction	94.4% (136)	71.8% (125)	70% (77)	79% (338)
Social interaction	5.6% (8)	28.2% (49)	30% (33)	21% (90)

Table 3 shows the variety of the containers R engaged with. Table 3 shows the containers that appeared in a total of more than 10 episodes over the three periods. Except for the open cloth box that contained R's toys, the puzzle box, the green block box, and the walker wagon's box that she frequently interacted with, we combined the small boxes, such as a food container or the shoebox, into the “box” category. As the green block box was introduced in the second period, there was no episode in the first period. A total of 48 containers were observed throughout the three periods. Chi-square tests showed significant differences in the frequency of occurrence among the periods for all items except for the box (Table 3).

**Table 3:** Frequently engaged containers in each period.

	**Period (number of episodes)**			
	**1st 7m23d**−**12m26d**	**2nd 12m29d**−**18m24d**	**3rd 19m1d**−**32m21d**	
	**(144)**	**(174)**	**(110)**	
Bag	1^**^	15	22^**^	χ^2^(2) = 28.75, *p* < 0.01
Block Box	0^**^	11	13^**^	χ^2^(2) = 16.74, *p* < 0.01
Bookshelf	3^**^	21^**^	7	χ^2^(2) = 11.87, *p* < 0.01
Box	6	10	5	χ^2^(2) = 0.46, *ns*
Cabinet	7	17^**^	4	χ^2^(2) = 5.15, 0.05 < *p* < 0.10
Cloth box	11^**^	17	28^**^	χ^2^(2) = 20.24, *p* < 0.01
Drawer (kitchen)	1^*^	12^**^	2	χ^2^(2) = 10.21, *p* < 0.01
Drawer (living)	0^**^	22	21^**^	χ^2^(2) = 27.34, *p* < 0.01
Pantry closet	15^**^	0^**^	1	χ^2^(2) = 27.05, *p* < 0.01
Puzzle box	16^**^	1^**^	1^*^	χ^2^(2) = 25.71, *p* < 0.01
Storage bench	59^**^	33	8^**^	χ^2^(2) = 42.72, *p* < 0.01
Tray	12^**^	2^*^	0^*^	χ^2^(2) = 17.86, *p* < 0.01
Walker wagon	9^**^	3	0^*^	χ^2^(2) = 10.19, *p* < 0.01

^*^p < 0.05.

^**^p < 0.01.

In the following sections, we examined the features of each period concerning the containers R frequently engaged with based on Table 3. We illustrated some of the characteristic cases for a better understanding.

### 3.2. The first period (7m23d−12m26d)

The containers R frequently engaged with included the storage bench, the pantry closet in the kitchen, the puzzle box, the walker wagon's box, and the tray for the wooden blocks (Table 3).

**Figure 3:**
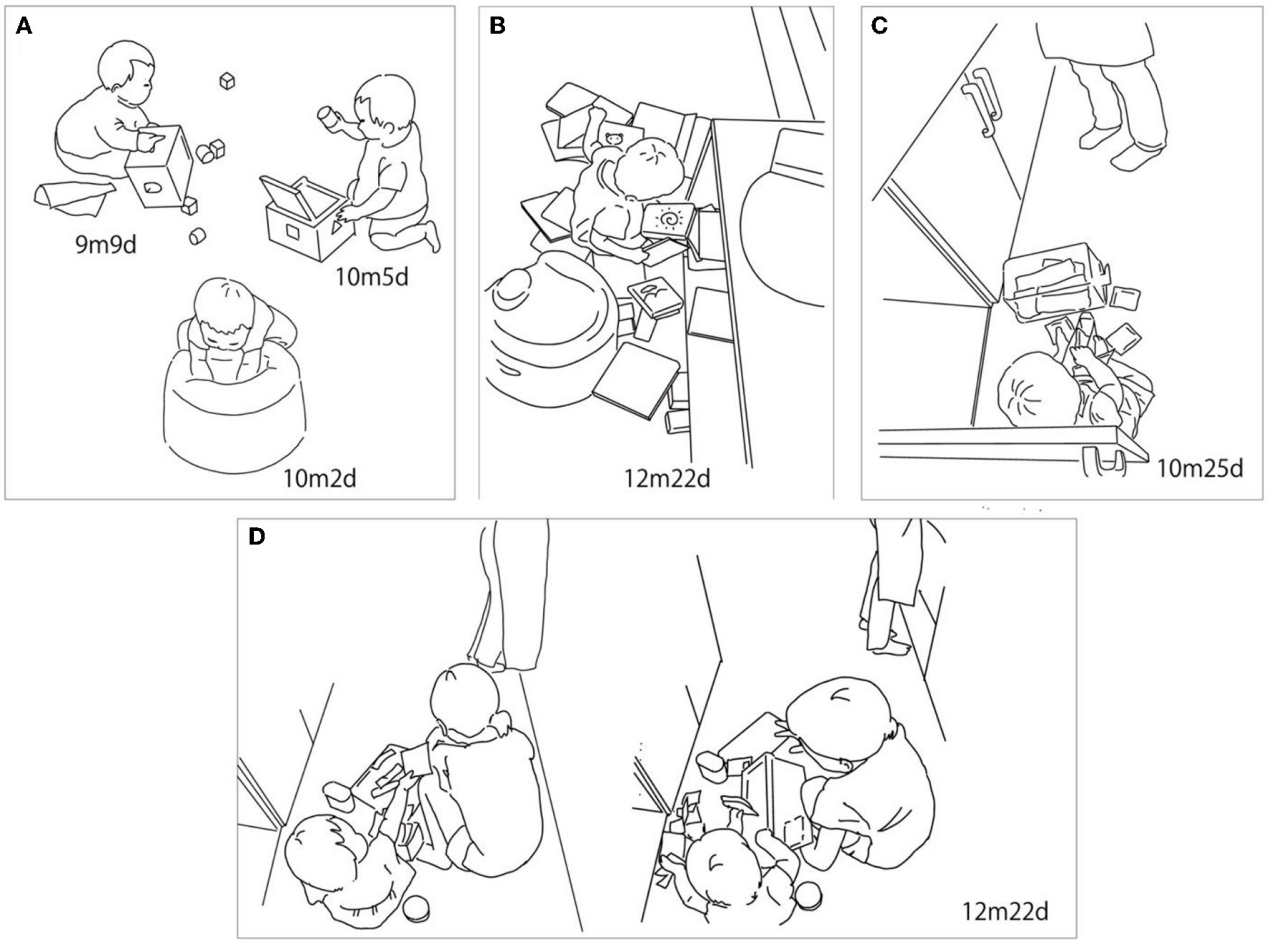
Infant–object interaction in the first period (7m23d−12m26d). **(A)** R engaged with blocks and small containers such as the puzzle box. She also put objects in a baby chair. **(B)** R pulled many books out of the storage bench. **(C)** R opened the cabinet in the kitchen, took out the food container, and put the teabags out. **(D)** R's sister gathered teabags that R had scattered, while R tried to hand one of the teabags to her sister. After her sister finished cleaning up and put the container in the cabinet, R grabbed it and spilled the objects inside again.

There were two features in this period. The first was putting objects in and taking them out of the small containers, such as the puzzle box (Figure 3A, 9m9d, 10m5d), the walker wagon box, and the tray. R's interactions with these containers in this period mainly included toy blocks. Putting blocks into and taking them out of the small containers such as the puzzle box was observed beginning at 9m 9d on the video. She also put blocks into the walker wagon box. Some blocks were organized in the block tray by her mother, and R picked them up from the tray to start playing. Along with the blocks, R also put small daily objects into concavities such as a baby chair (Figure 3A, 10m2d).

The second feature was successively pulling out many objects from the containers, such as the storage bench (Figure 3B, 12m22d), the pantry closet, and the cabinet in the kitchen (Figure 3C, 10m25d). There were picture books stacked on the storage bench; in fact, R pulled many books out of the storage bench successively. All of the books she spread out were occasionally picked up by her mother, but she repeatedly pulled them out again, even on the same day. She also pulled daily items out from the pantry closet in the kitchen when her mother opened it. Beginning at the end of 10 months, R started exploring the kitchen and learned to open the cabinet door by herself, after which she took the food container out and spread out the things that were inside, such as teabags (Figure 3C, 10m25d). These episodes did not include carrying, so objects were scattered in place.

R's mother cleaned the objects that R scattered after she left. However, some of the activities involving putting things in and taking them out included social interaction. In Figure 3D, once her sister collected the teabags R scattered, R tried to hand one to her sister. After her sister finished putting things away and tried to put the container into the cabinet, R uttered sharply and grabbed the container to pull them out again. After taking them out, R began to put them into the container (12m22d).

### 3.3. The second period (12m29d−18m24d)

The containers R frequently engaged with included the cabinet, the drawers, and the small bookshelf in the living room (Table 3). She also engaged with bags and the green block box that appeared at 14m15d. In this period, R still frequently pulled the books out of the storage bench. She also pulled books off of the bookshelf.

**Figure 4:**
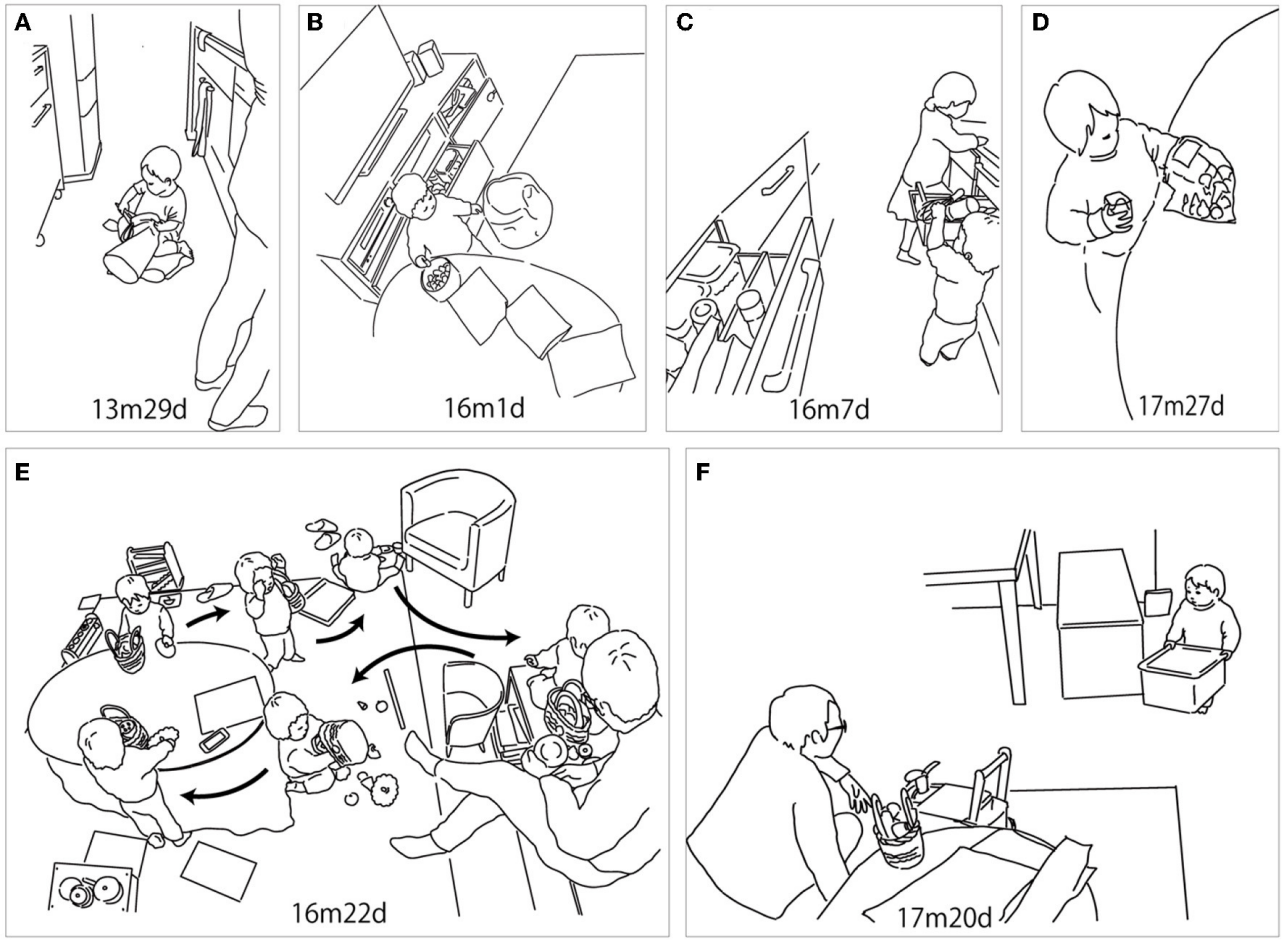
Infant–object interaction in the second period (12m29d−18m24d). **(A)** R put the kitchen utensils into the bag that she got in the living room. She then carried it to the living room. **(B)** R took the round box (crayon box) from the drawer, put it on the table, and took one crayon out of the box to draw on the paper. **(C)** R took a bottle out of the drawer and tried to push it into the other drawer behind her. **(D)** R took crayons out of the plastic bag and put them into the cup. **(E)** R carried toy cups and plates using the basket bag. R took the objects out, then put them in the bag again and carried them around the room. **(F)** R took the green block box from the storage bench and brought it to her father to take the blocks out to play with him.

There were three features in this period. The first, putting objects in and taking them out, was nested into carrying by using containers; more than half of the scenes included carrying objects. She occasionally used bags or other containers to carry objects. Figure 4A shows R carrying kitchen utensils using the bag that she got in the living room (13m29d). Figure 4E shows her carrying toy cups and plates using the basket bag. In this case, R took objects out, put them in the bag again, and carried it around the room. It lasted for more than 15 min (16m22d).

The second feature was bringing the containers for the next activity. In Figure 4B, R took the round box (crayon box) from the drawer, put it on the table, and took one crayon out of the box to draw on the paper (16m1d). In Figure 4F, R took the green block box out of the storage bench and brought it to her father to take the blocks out and play with him (17m20d).

The third feature was putting objects into another container. R tried to take objects out and fit them in another place. In Figure 4C, R removed a bottle from the drawer and tried to push it into the other drawer behind her (16m7d). In Figure 4D, R took crayons out of the plastic bag and put them into the cup (17m27d).

### 3.4. The third period (19m1d−32m21d)

The containers R frequently engaged with included the bags, the open cloth box, the drawer in the living room, and the green block box (Table 3). Carrying was common, as 70% of the episodes included carrying.

**Figure 5:**
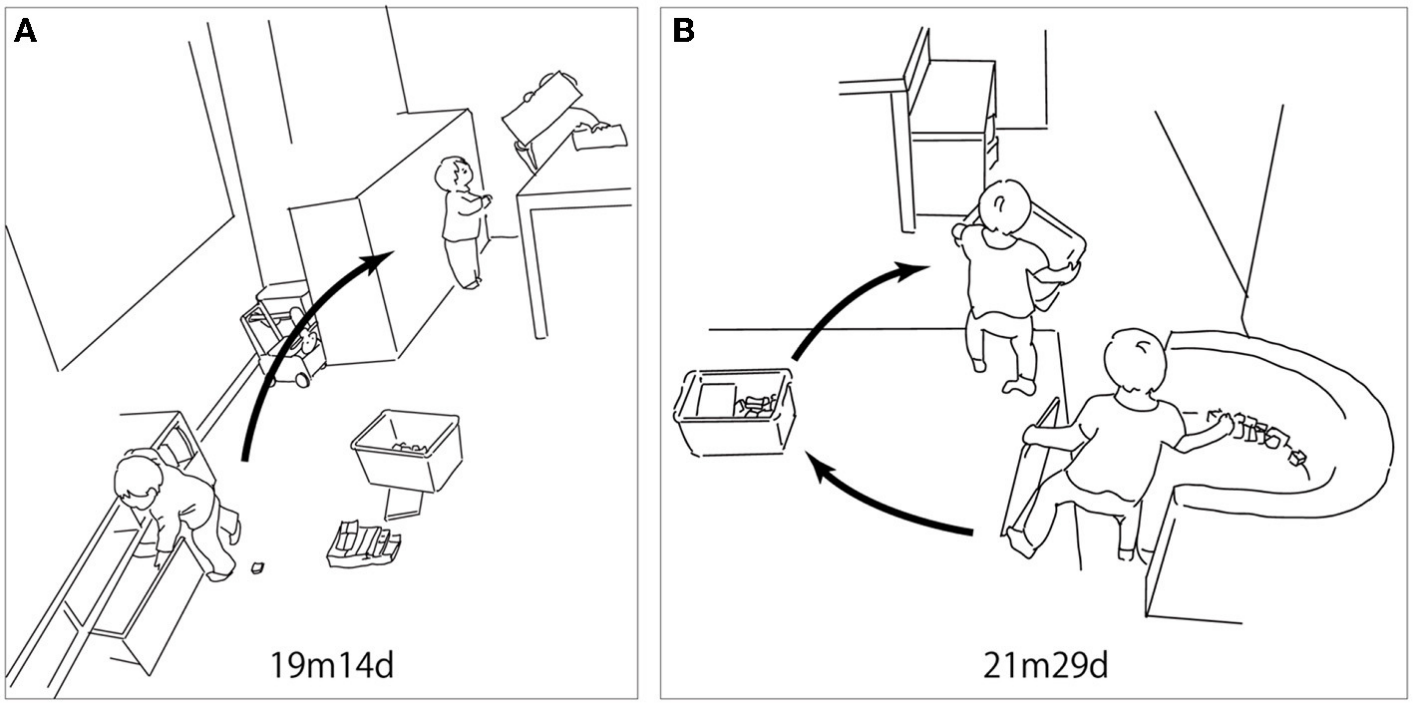
Infant–object interaction in the third period (19m1d−32m21d). **(A)** R took a piece of paper out of the drawer that held her toys inside; she handed it to her sister, who was already drawing on the table, to join the drawing. **(B)** R was playing with her sister using blocks. Once her sister started to put the blocks away, R took some blocks out. Her sister grumbled to R. Just after her sister left the scene, R grabbed the blocks left behind and hurried to put them into the box. She cleaned all of the blocks and carried the box to the storage bench where it belongs.

There were two features in this period. The first was that objects taken out of the container became more specific to the context. Though R frequently pulled out many books from the storage bench or bookshelf in the previous two periods, such activities became rare in the third period. In Figure 5A, R took a piece of paper out of the drawer holding her toys and handed it to her sister, who was already drawing on the table, so that she could join in the drawing (19m14d). In another case, after her mother played a cartoon on TV, she rushed to the open cloth box that contained her toys and took the plush toy resembling a TV character out of the box (21m14d).

The second feature was bringing out the container before the activity and putting things away after a play session. In Figure 5B, R was playing with her sister using blocks. Once her sister started to put the blocks together and put them into the box to clean them up, R put some blocks out. Given this, her sister was not happy with R and grumbled at her. Just after her sister left the scene, R grabbed the blocks left behind and hurried to put them into the box. She picked up all of the blocks and carried the box to the storage bench where it is kept (21m29d). In another case, R pulled a bag out of the cloth box as well as other toys from the drawer and put them on the table to play, which included combining items by herself. After playing with them for several minutes, she carried the bag to the cloth box and put some toys into the drawer (24m28d). At the end of this period, there was a case wherein she asked for help from others. After she played with the toy cups and plates for a while, she started to put them into the basket and the box. She could not close the toy set by herself, so she asked her sister for help. Her sister closed it, and R took the set and put it in the corner of the room where it should be (32m21d). In the first case (21m29d), R's mother told R's sister to put the blocks away. The mother would talk to R when she was cleaning up, saying, for example, “I'm putting blocks away.” She did not explicitly instruct R to put objects away.

## 4. Discussion

This study aimed to examine the development of organized object interaction in the context of everyday activities. We observed a child's development of object interaction from the age of 7 to 32 months old by focusing on the scenes in which she put objects in and took them out of containers. In the first period covering 7–12 months of age, putting things away and taking them out appeared early, at 9 months old; specifically, this was accomplished by using small containers such as the puzzle box. After her exploration area expanded, she removed daily goods from the kitchen cabinet and the pantry closet; she also liked to pull many books from the storage bench. Putting many things in and taking them out itself emerged as a style of play. In the second period covering 13–18 months of age, after acquiring the skill of walking, she carried objects by using bags. Therefore, what emerged at this point were the actions of putting objects in and taking them out that are embedded in the locomotion and preparation of containers of toys before the activity. She also started to explore affordances between objects and containers by taking objects out of one container and putting them into different containers. In the third period covering 19–32 months of age, taking as many objects out as possible became rare, and when the objects were pulled out, it was more specific to the context of the situation. Thus, bringing the container before the activity as well as putting things away after a series of plays was observed.

### 4.1. Development of organized infant–object interaction and the development of anticipation

**Figure 6:**
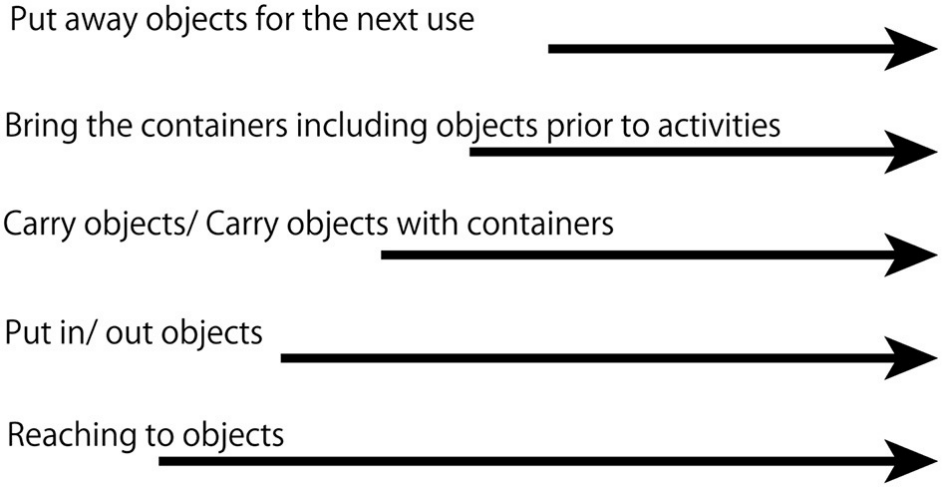
Hierarchical structure of the development of organized behavior in putting away objects.

In this study, R's organized interaction with objects developed based on the experiences of putting objects in and taking them out of containers in an exploratory manner. We propose a developmental theory in which previously acquired actions are the basis for further exploration (Figure 6). Previous studies have revealed the exploratory nature of infant behavior (Adolph et al., 2012; Cole et al., 2015; Hoch et al., 2018). We showed the process of how exploratory behavior converges into organized behavior. As shown in Figure 6, the exploratory actions are supported by previously acquired actions. For example, reaching, which was acquired before the observation period of this study, worked as a basis for the action of putting objects in and taking them out of containers. Moving objects into and out of containers in an exploratory manner leads to carrying objects by using containers such as a bag. Subsequently, carrying containers leads to the development of knowledge of functionally different places. Thus, there is a hierarchical structure to organized activities. The child did not continue with the exploratory activities that were observed in the first period for the entire observation period, and the activities eventually converged into the scope of the routine of daily life. Thus, exploration can be the basis for exploitation.

The salient difference between the first and second periods was walking and carrying. The object interaction was embedded in carrying, and R started using bags to carry objects. Taking objects out became more anticipatory. One action of taking something out was nested in a preliminary action of taking something out; before drawing, she removed crayons from the crayon box that was inside the drawer. This suggests that experiencing combining objects with containers and bringing them to another place leads to the development of organized object interaction. Previous studies revealed that walking and carrying change behavior in regard to relating to others (Karasik et al., 2012, 2013). Therefore, not only does carrying objects affect social development, but it may also influence cognitive development relating to the understanding of the temporal structure of events generally understood as a script of daily routines. This refers to knowledge about a certain sequence of daily behavior. For example, for a certain event to occur, such as drawing on paper, the action of taking the crayon box out must precede the action of taking a crayon from the box.

In the third period, putting objects away after play activities emerged; bringing containers that include objects that are necessary for the upcoming activity is the preparation. There is anticipation for the upcoming activity. This type of behavior was observed beginning in the second period. In the third period, which was around the age of 2 years old, R started putting objects into a container and returning it to their original place. Regarding this matter, there is longer anticipation for the next use. Even in the first period, she took things out and put them in the same container. However, the scale for anticipation was short, as we have illustrated in 12m22d (Figure 3).

### 4.2. Social environment for the development of object interaction

Social interaction also leads to the development of organized object interaction. In the first period, in some cases, R took out objects and handed them to others. In the second period, she brought the block box to start playing with others. In the third period, she took objects out to join the ongoing activity that others had begun. In the case, we illustrated in Figure 5B, her sister's attempt to clean up the blocks was a chance for her to perceive the end of the play session. R changed the action from taking the blocks out to putting them back in the box (21m29d). Sharing the same objects with others also leads to the decision of what to do with the objects.

The perception of the affordances of objects and containers is learned in a social environment. Observing what others do affected the way R engaged with objects and containers. Activities such as cleaning up toys have been studied from the perspective of social rules or compliance with the parents (Power and Parke, 1986; Gralinski and Kopp, 1993; Kochanska et al., 2001). In particular, Kochanska et al. (2001) investigated children's and mothers' behavior longitudinally in a laboratory playroom and found that compliance increased from 14 to 33 months of age. Our study suggested that along with cooperation with others and rule-following, everyday experiences, exploring affordances between objects and containers and using them in different places, and sharing the objects with others influenced this behavior development.

Though the behavior of pulling many objects out and spreading them out decreased in the third period, this behavior from R led others to be involved. Orrmalm (2020) used ethnographic means to describe when infants spread things out at home and pointed out that “the flows of things” would shape how objects are ordered in the home by adults. Orrmalm (2020) also discussed that objects with which infants engaged were multiple not only in the sense they were many but also in the sense they could be understood as belonging to multiple locations in the home, having multiple functions or usages, or belonging to multiple people. Not only may carrying objects boost the change in relationships with others, but infants also discover different functions of the same objects at many places and actively contribute to changing their living environment.

### 4.3. Longitudinal observation in the daily environment

We focused on one child, which enabled us to understand the longitudinal change in behavior with the same containers or furniture. The behavior developed into an organized one based on exploration. We revealed what a child did with objects in their daily life, and it led us to consider the effect of interaction with a variety of objects around them in their daily environment. Herzberg et al. (2021) examined infant–object interaction at home and showed the variety of objects that infants engaged with. The study indicates that brief but frequent variable interactions with objects may be conducive to learning object properties and function, motor skill acquisition, and influence cognitive, social, and language development. Our basic idea that frequent object interactions with a variety of objects in the daily environment would become an important role in social and cognitive development followed the results of Herzberg et al. (2021). However, we focused less on the variety of objects and more on the same containers to reveal the developmental change of object interactions in the daily environment.

Recent studies on infant behavioral development focus on the developmental cascade; as such, a certain developmental change, such as the acquisition of walking, can instigate a wide range of behavioral development (Adolph and Robinson, 2015; Adolph et al., 2018). By observing the natural object interactions at home longitudinally, our study showed how walking, carrying, interacting with objects, and interacting with objects and people changed the opportunities for behavior. This may clarify the role of everyday experiences in behavior development.

### 4.4. Limitations and future research

Our descriptions of the child's natural object interactions provide opportunities to consider behavioral development as an integrated type of development while not assuming the divided fields such as cognitive, social, or motor development. However, our study is a single-case study, so it is difficult to examine the hypothesis. More cases are necessary to understand development variations. Adding international cases would also help to understand the cultural differences or the common features among the cultures. Parents' attitudes toward clean-up may also affect how children learn to organize objects. Correlates with intellectual development can also be considered.

## Data availability statement

The datasets presented in this article are not readily available because of privacy restrictions. Requests to access the datasets should be directed to the corresponding author.

## Ethics statement

The studies involving human participants were reviewed and approved by the University of Tokyo. Written informed consent to participate in this study was provided by the participants' legal guardian/next of kin.

## Author contributions

CN, HN, HY, and KK contributed to the conception of the study. CN and HY collected and coded the data. CN wrote the draft. HN contributed methodology. CN and KK contributed to the funding acquisition. All authors contributed to the article and approved the submitted version.
